# How molecular motors work – insights from the molecular machinist's toolbox: the Nobel prize in Chemistry 2016

**DOI:** 10.1039/c6sc04806d

**Published:** 2016-11-21

**Authors:** R. D. Astumian

**Affiliations:** a Department of Physics , The University of Maine , Orono , ME 04469 , USA . Email: Astumian@maine.edu

## Abstract

The Nobel prize in Chemistry for 2016 was awarded to Jean Pierre Sauvage, Sir James Fraser Stoddart, and Bernard (Ben) Feringa for their contributions to the design and synthesis of molecular machines. While this field is still in its infancy, and at present there are no commercial applications, many observers have stressed the tremendous potential of molecular machines to revolutionize technology. However, perhaps the most important result so far accruing from the synthesis of molecular machines is the insight provided into the fundamental mechanisms by which molecular motors, including biological motors such as kinesin, myosin, F_o_F_1_ ATPase, and the flagellar motor, function. The ability to “tinker” with separate components of molecular motors allows asking, and answering, specific questions about mechanism, particularly with regard to light driven *vs.* chemistry driven molecular motors.

## Abstract 2

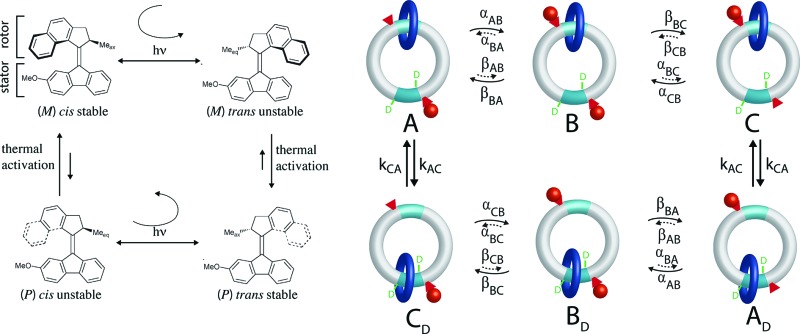
The Nobel prize in Chemistry for 2016 was awarded to Jean Pierre Sauvage, Sir James Fraser Stoddart, and Bernard (Ben) Feringa for their contributions to the design and synthesis of molecular machines.

## 1 Introduction

Molecular motors drive almost all significant biological processes, yet very few if any man made technologies exploit controlled molecular motion. The work for which the 2016 Nobel prize in chemistry was awarded has begun to change this state of affairs by presenting inspired synthetic routes to facilely create a host of interlocking structures known as catenanes^1^ and rotaxanes,^2^ and directional rotors^3^ that work with, rather than against, ubiquitous thermal noise.

At first, many systems operating as simple back and forth molecular switches were proudly proclaimed to be “molecular motors”. Eventually, conceptual advances^4,5^ that showed how omnipresent thermal noise can be exploited at the nano-meter scale filtered into the synthetic chemistry community. This insight led to synthesis of molecular machines with the ability to use external energy to power the cyclic motion necessary for creation of true molecular motors. Detailed study of different mechanisms – energy ratchets and information ratchets^6^ – by which this occurs has led to important gains in understanding how biomolecular machines work. Specifically, the “power-stroke”, presented as the mechanism by which most ATP and ion gradient driven molecular motors work in almost all leading textbooks of biochemistry and cell biology, is shown to be just plain wrong for these chemically driven motors. Instead, the key mechanism for molecular motors driven by a catalysed reaction such as ATP hydrolysis or ion transport is chemical gating in which the specificity for substrate *vs.* product depends on the state of the molecular motor, a mechanism known as an information ratchet.^6^

## 2 Molecular rotors and motors

Consider two very different designs for a molecular rotor, one, a light driven rotor from Ben Feringa's group,^7^ and the other an autonomous rotor driven by catalysis of a chemical reaction from David Leigh's group.^8^

### Light driven rotor

**Fig. 1:**
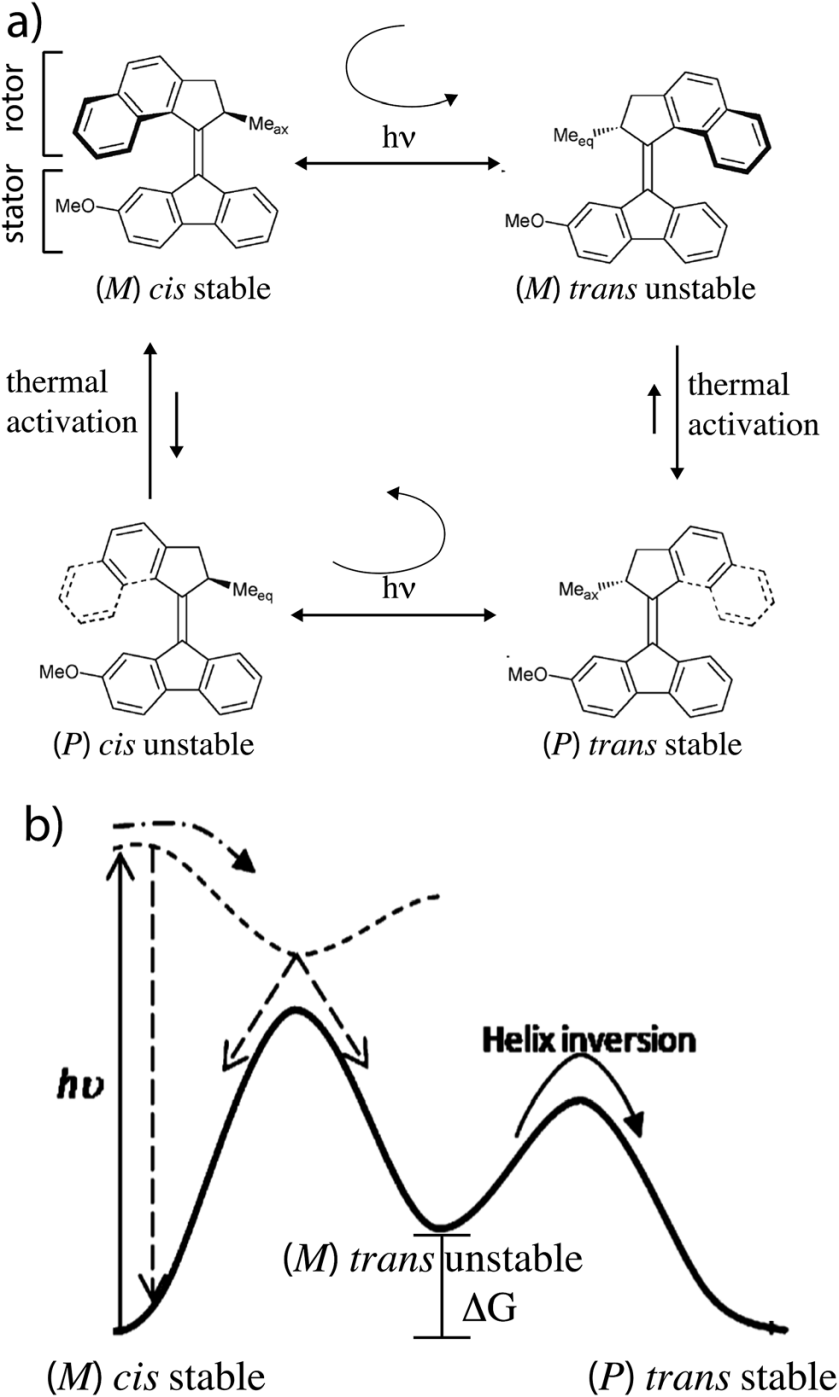
(a) Light driven molecular motor cycle designed by Ben Feringa and colleagues. The design is based on over-crowded alkenes, where the notation (M) or (P) denotes left (Minus) or right (Positive) helicity. For convenience we designate the bottom part of the molecule as the “stator” and the top part as the “rotor”. The subscript on the methyl group (Me) at the chiral carbon atom of the rotor denotes whether the orientation of the methyl is axial (ax) or equatorial (eq). (b) Ground (solid curve) and excited state (dashed curve) energy surfaces for motion of the rotor relative to the stator of the motor in (a).

The light driven motor described by Feringa and colleagues^7^ uses optical energy to populate an unstable state by a directional rotation followed by a thermal relaxation to a stable state in a cyclic fashion resulting in completion of a rotation about a carbon–carbon double bond. In Fig. 1 absorption of a photon by the stable (ground) states (M) *cis* isomer causes clockwise rotation as the molecule is promoted to an unstable (excited) state (M) *trans* isomer. This is followed by thermally activated helix inversion to the stable (P) *trans* isomer. Absorption of a photon by the (P) *trans* isomer leads to clockwise *trans*–*cis* isomerization to the unstable (P) *cis* isomer followed by thermally activated helix inversion to the starting (M) *cis* isomer. In the excited state the rotor freely rotates on one side of the plane of the stator but is hindered from helix inversion. In the ground state the barrier for helix inversion is lower than that for rotation about the carbon–carbon double bond, with the net effect being continuous rotation of the molecule when illuminated.

The stability difference, Δ*G*, between unstable and stable states is determined by the intramolecular strain when the methyl group on the rotor is in the equatorial *vs.* axial positions. It is key that this Δ*G* be relatively large to ensure directional rotation. The relative likelihood of completing a clockwise *vs.* counter-clockwise rotation is

1 *r* = e^2Δ*G*^

where Δ*G* is, as are all energies in this paper, expressed in units of the thermal energy.

While early light driven motors^3^ were driven by cycles of illumination – heating – cooling – illumination, recent motors have been designed to allow for continuous operation at MHz rotation frequencies at a fixed temperature.^7^ Similar ideas arise in stochastic conformational pumping mechanisms^9^ ^*a*,*b*^ where time periodic external driving induces net cycling if some transitions involve a significant Δ*G*. The mechanical process in which the energy Δ*G* is dissipated in the environment is very reasonably termed a power stroke. Let us compare the light driven motor that requires a power stroke with a motor driven by catalysis of a chemical reaction.

### Chemical catalysis driven rotor

Most biomolecular motors use energy from ATP hydrolysis or transport of protons from high to low electrochemical potential across a membrane rather than from light or from external modulations. A longstanding challenge has been to design a synthetic motor that similarly uses chemical energy from a catalyzed reaction to drive directed motion. This goal has recently been achieved^8^ by David Leigh's group using catenanes, a type of the mechanically interlocked molecules held together by mechanical bonds for which Jean Pierre Sauvage^1^ and Sir Fraser Stoddart^2^ were awarded the Nobel prize. The rotor described by Wilson *et al.* ^8^ incorporates the main features of biological molecular motors – chemical catalysis, gating of the binding and release of substrate and product depending on the mechanical state of the motor, and conformational changes involving mechanical motion.

The molecular motor, a [2]catenane, comprises two interlocked rings of different sizes. The small blue ring is free to rotate relative to the larger ring, shuttling between the two recognition sites (shown in aqua) on the larger ring, one of which is labelled by deuterium. There are two catalytic sites (hydroxyl groups) represented by the small red cones, one near each recognition site, that catalyze the conversion of Fmoc-Cl to dibenzofulvene.

**Fig. 2:**
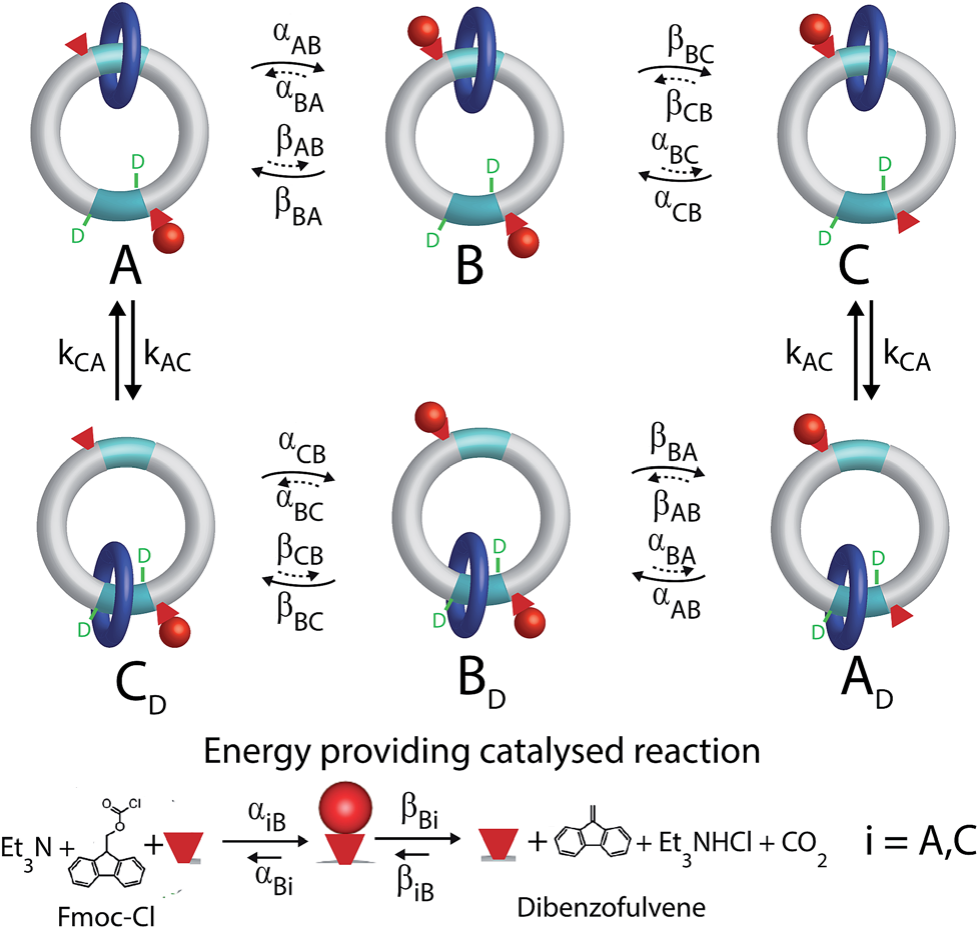
Kinetic cycle for the molecular motor described by Wilson *et al.* ^8^ The deuterated recognition site serves as a fiduciary marker for determining directionality but does not otherwise influence the kinetics or thermodynamics of the motor.

A kinetic cycle for the mechanism of the motor is shown in Fig. 2. The energy-releasing reaction is the conversion of the protecting group 9-fluorenylmethoxycarbonyl chloride (Fmoc-Cl) to dibenzofulvene. The free-energy released by this reaction is greater than that released by ATP hydrolysis. The key point is that the addition of the protecting group occurs as Fmoc-Cl and the removal of the protecting group is as dibenzofulvene, processes that are not the microscopic reverses of one another. The description of the catalytic conversion of Fmoc-Cl to bibenzofulvene is shown as a Michaelis–Menten catalytic mechanism at the bottom of the Fig. 2, thus emphasizing that the hydroxyl groups function as catalysts even though the focus is on the effects of the small-molecule reagents on the large moving ring.

It is very natural to imagine that, by analogy with Feringa's light driven motor, clockwise rotation of the blue ring from the perspective of the reader could be enforced by designing the system with a repulsive interaction between the bulky Fmoc protecting group (red sphere) and the dark blue ring, thereby engineering a “power-stroke” in the direct mechanical transitions such that *k*_CA_ > *k*_AC_ and hence with 
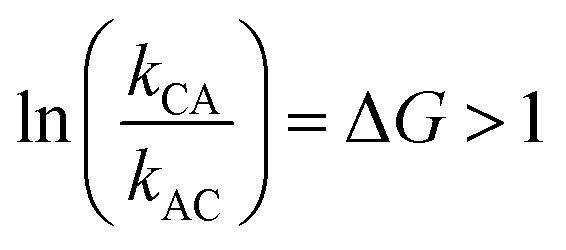
 driving the transition C → A.

This intuitive idea however is wrong – the dark blue ring shown in Fig. 2 actually rotates (from the perspective of the reader) counter-clockwise irrespective of whether the interaction between the small blue ring and the Fmoc protective group is attractive (Δ*G* < 0) or repulsive (Δ*G* > 0). The directionality arises because the rate of attachment of the protecting group to the catalytic site is faster when the blue ring is at the distal rather than proximal recognition site (*α*_CB_ > *α*_AB_). The rate of cleavage of the Fmoc group as dibenzofulvene, on the other hand, is nearly independent of the position of the ring (*β*_BA_ ≈ *β*_BC_). These two kinetic conditions on the rate constants reflect the fact that installing the protecting group as Fmoc depends on the location of the dark blue ring, but that removing the protecting group as dibenzofulvene does not. The net effect of these two kinetic conditions is to make the transition C → A less likely than the transition A → C, a seemingly slight bias which is nevertheless enough to provide directionality to the rotational motion although the experimentally observed velocity is very small.

It is essential at this point to make a very important distinction between the autonomous chemically fuelled motor of Wilson *et al.* ^8^ and previously described “chemically driven” molecular motors and pumps which were driven by external changes of the chemical environment – redox potential, pH, substrate concentration, *etc.* Such externally driven motors can, and for the most part do, work by a power stroke mechanism similar to the light driven motor described by Feringa and colleagues^7^ and the biological pump driven by a fluctuating electric field described by Astumian *et al.* ^9^ ^*a*^

The motor described by Wilson *et al.* ^8^ on the other hand works under a single reaction condition where substrate (Fmoc-Cl) associates and product (dibenzofulvene) dissociates because the chemical potential of substrate is large and that of product is small. This reproduces a major feature of ATP hydrolysis driven biomolecular motors. Autonomous motors driven by catalysis of a chemical reaction cannot operate by a power stroke mechanism, and instead function as information ratchets^6^ where the key mechanistic design principle is the kinetic discrimination of the rate of the chemical reaction depending on the mechanical state of the motor by which *α*_CB_ > *α*_AB_.

In Fig. 2 rate constants for cleavage of the protecting group as Fmoc, and attachment as dibenzofulvene are shown, even though the rates for these processes are very small under the experimental conditions. This is done to allow for self-consistent examination of the kinetics and thermodynamics of this molecular motor.^8,10^ The rate constants must obey the constraint2
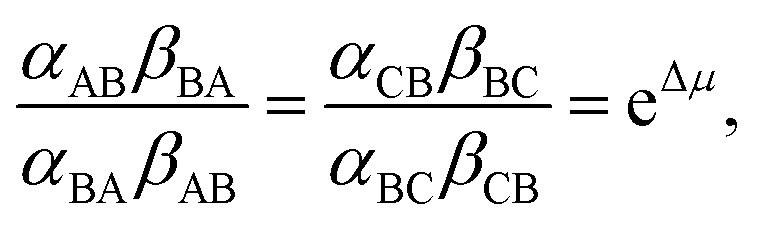
where 
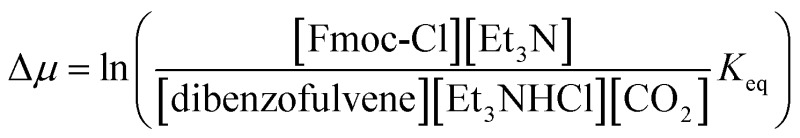
 and *K*_eq_ is the equilibrium constant for the catalyzed reaction. An additional constraint3
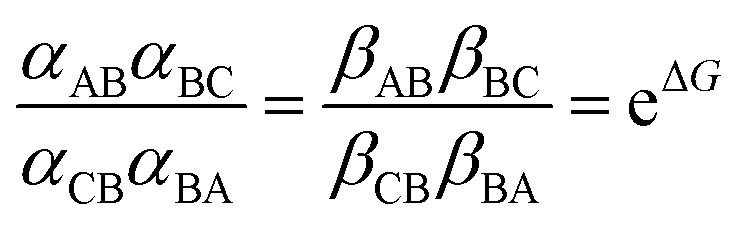
must also hold. The coefficients *α*_BA_, *β*_BA_, *α*_BC_, *β*_BC_ are first order “off” rate constants and the ratio between any two of them is independent of the free energies of the states A, B, and C and can be written as the exponential of the difference between two activation energies. The coefficients *α*_AB_, *β*_AB_, *α*_CB_, *β*_CB_ on the other hand are pseudo-first order rate constants into which the bulk concentrations [Fmoc-Cl] and [Et_3_N], for the *α*'s, and [dibenzofulvene], [Et_3_NHCl], and [CO_2_], for the *β*'s, have been subsumed. The two relationships eqn (2) and (3) are thermodynamic identities that follow from the principle of microscopic reversibility.^11^

The directionality of the molecular motor in Fig. 1 is given by the ratio of the products of the net clockwise and counter-clockwise rate constants,4
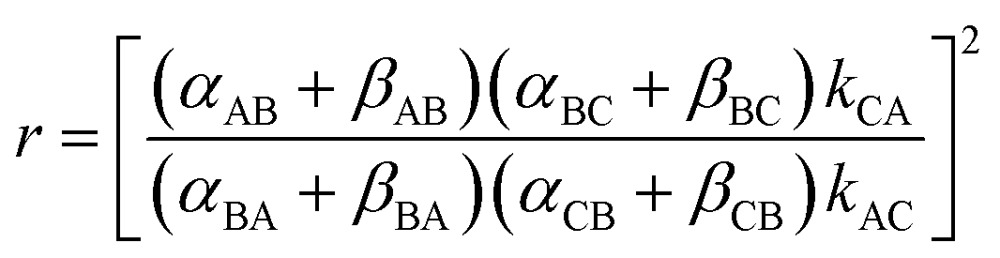
when *r* > 1 the blue ring rotates clockwise, and when *r* < 1 the blue ring rotates counter-clockwise, from the perspective of the reader.

A regrettable tendency in many recent papers is to point out that chemically driven motors “operate far from equilibrium” and then assign rate constants *ad libitum* for mathematical convenience rather than for thermodynamic consistency. This approach often leads to plausible but incorrect conclusions. As an example, consider the assignment *α*_AB_ = *α*_CB_ = *β*_BA_ = *β*_BC_ = *γ* e^*μ*/4^ and *α*_BA_ = *α*_BC_ = *β*_AB_ = *β*_CB_ = *γ* e^–*μ*/4^, where *γ* sets the time scale for the chemical transitions relative to the mechanical transition. With these expressions we immediately derive as the ratio of clockwise to counter-clockwise net rate constants the expression *r* = e^2Δ*G*^, seeming to suggest a critical role for the “power-stroke” just as for light driven motors. However, while this assignment for the rate constants is consistent with eqn (2), it is not consistent with eqn (3) and is thus fundamentally incorrect, being tantamount to ignoring both the steric interaction by which *α*_CB_ > *α*_AB_, and any energetic interactions by which *k*_CA_ ≠ *k*_AC_ and which must be reflected in the *α*'s and *β*'s according to eqn (3). The negligibility of energetic interactions in the chemical rate constants is absolutely impossible even very far from equilibrium as pointed out first in the context of ion pumps.^9^ ^*a*^

When we carry out the slightly more mathematically complex imposition of the constraints of both eqn (2) and (3) we instead obtain the correct expression5
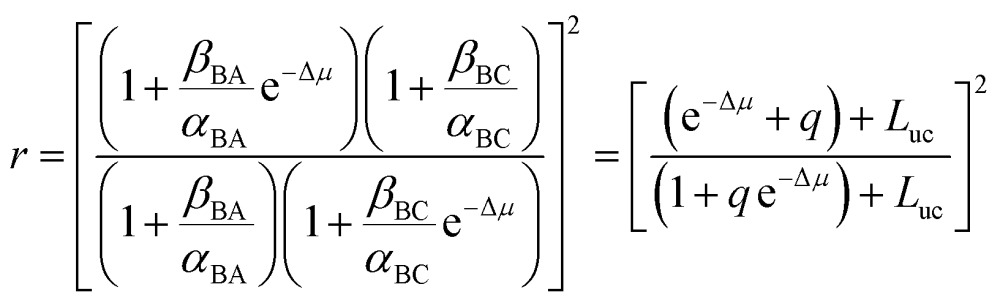
where *q* = *α*_BA_ *β*_BC_/*α*_BC_ *β*_BA_ is a ratio of “off” rates and parametrizes the chemical gating. Thus we see that *r* is independent of Δ*G*. The term *L*_uc_ arises from un-coupled cycles^12^ and setting *L*_uc_ = 0 represents the optimal directionality of the motor. Not surprisingly, if Δ*μ* = 0 then *r* = 1. More revelatory is the fact that if *q* = 1 then *r* = 1 irrespective of the value of Δ*μ*, *i.e.*, if there is no chemical gating there is no coupled rotation despite continual chemical dissipation. Using the constraints eqn (2) and (3), with *α*_CB_ > *α*_AB_ and *β*_BA_ ≈ *β*_BC_ we have for the motor in Fig. 2 that *q* < 1 and hence that *r* < 1, irrespective of Δ*G*.

There is a clear presumption in the literature that the Δ*G* for the mechanical stroke must be positive in the direction of motion. Howard comments that “If the structural states differ by a distance *d*, then the power stroke can do work against an external loading force *F* provided that *F*·*d* ≤ Δ*G*, where Δ*G* is the decrease in free energy between the two chemical states”.^13^ and Eisenberg and Hill claim that the maximum efficiency can be no higher than Δ*G*/Δ*μ*, where Δ*μ* is the free energy released by the driving chemical reaction.^14^ These assertions regarding the power stroke, based on the incorrect assumption that the “power stroke” must be energetically downhill (exergonic), are just wrong for chemically driven motors. In fact, the thermodynamic properties of a chemically driven motor – efficiency, stoichiometry, stopping force – do not depend on Δ*G* at all.^12,15^ Perhaps having different stabilities for A and C affords a kinetic advantage? This idea, too, is incorrect. While the rate of the mechanical transition may indeed be enhanced when Δ*G* ≠ 0, the net rate of the chemically driven transitions between A and C through state B is inexorably decreased when the free energies of A and C are different from one another. In fact, the rate of rotation is optimized when the basic free energies of all states are identical, consistent with Jeremy Knowles theory for evolutionary optimization of enzyme catalytic function.^16^

Now consider the case that *q*, e^–Δ*μ*^ ≪ 1 so that *q* e^–Δ*μ*^ can be neglected in eqn (5). We recognize two distinct regimes of coupled transport.^12^ When gating is very strong (*q* ≪ e^–Δ*μ*^) the motor operates in the thermodynamic control regime, also known as the tight-coupling regime, in which every conversion from substrate to product is accompanied by a counter-clockwise rotation, and application of torque sufficiently strong as to cause clockwise rotation induces conversion of product to substrate. An example is the F_1_ ATP synthase where ATP hydrolysis drives rotation in one direction. When an external torque is applied to force rotation in the opposite direction ATP is synthesized.^17^

In contrast, when e^–Δ*μ*^ ≪ *q* the motor is regulated by the kinetic gating. In this regime the application of a sufficiently strong torque to cause a motor to move backward results in an increase in the rate of conversion of substrate to product, not in the conversion of product to substrate. This latter behaviour, surprising to many in the field of biological motors, was predicted by Astumian and Bier in 1996 (ref. 18) and demonstrated experimentally for kinesin by Nishiyama, Higuchi, and Yanagida^19^ and by Carter and Cross^20^ in 2002 and 2005, respectively.

## 3 Prospective

**Fig. 3:**
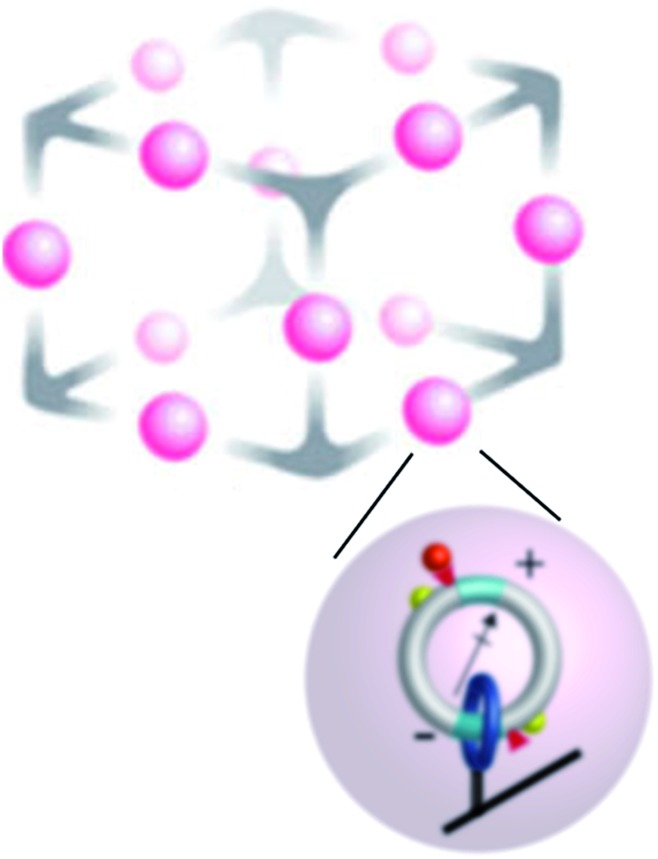
Schematic approach to “smart materials” that can interact with, and mediate energy exchange between, several modalities of energy input from the environment. The upper schema illustrates a single unit cell of a large periodic array, while the lower scheme shows an individual chemically driven molecular rotor similar to that describe in Fig. 2. Here, the catenanes localized on a metallic–organic framework (MOF) have dipole moments by which they can interact with an applied electric field, and chemical catalytic sites by which they interact with substrate and product in the environment. The yellow hemispheres are “molecular speed bumps” by which the height and position of the kinetic barrier to rotation can be engineered. In principle, energy can be transduced between an applied time-dependent electric field and the catalysed reaction in either direction. Cooperative interactions between the individual catenane rotors can give rise to very complex behaviour.

Molecular motors can be designed to interact with a variety of inputs – light, chemical reactions, electric fields, external changes in pH and redox potential, *etc.* – and to provide a means for energy transduction between these inputs. Finding the right environment to harness these possibilities has, however, not been easy since many possible tasks for molecular machines require spatial and/or temporal coordination between numerous individual motors. One approach to achieving this goal involves incorporating molecular motors in metallo–organic frameworks^21–23^ (MOFs) as shown schematically in Fig. 3. This setup affords the possibility of using molecular motors to perform molecular tasks. In principle, the catalytic function performed by the two active sites shown could be designed at will, here described generically as S → P. In the example shown in Fig. 3 an electric dipole is incorporated in the design of the elementary molecular motors based on that of Wilson *et al.* ^8^ Then an applied oscillating electric field 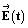 influences the motion^24,25^ and can drive the catalysed reaction away from equilibrium, maintaining a steady-state chemical potential difference^24,26^ 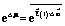 where 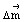 is the dipole moment difference between the states. Such coupled devices could in principle pave the way to new synthetic approaches for high energy (and high value) compounds as well as for new sensors.

There are of course approaches other than those based on a synthetic chemistry perspective, including those grounded in surface science^27–29^ and self-organized assembly^30^ in soft condensed matter.

## 4 Conclusions

The very different design principle necessary for a motor driven by chemical catalysis – gating – in comparison to that for a light driven or externally driven motor – power stroke – highlights the importance of microscopic reversibility for understanding the mechanism of chemically driven molecular motors.^31^ ‘In the IUPAC definition^32^ of the principle of microscopic reversibility, “In a reversible reaction, the mechanism in one direction is exactly the reverse of the mechanism in the other direction. This does not apply to reactions that begin with a photochemical excitation”, light driven processes are explicitly excluded from the constraint of microscopic reversibility’.

This is because in the absorption of a photon, and its reverse – stimulated emission – there is conversion between energy in one degree of freedom, the photon, and another single degree of freedom, the energy of the photo-chemically active molecule. In contrast, a thermal activation process, including binding of a high free-energy substrate, involves conversion between many degrees of freedom in the bath and only one (or perhaps a few) degrees of freedom in the molecule, irrespective of how far from equilibrium the reaction may be.

The importance of microscopic reversibility is also well illustrated in the context of a synthetic molecular pump.^33^ When driven by external changes of the redox potential it is essential that the energy of an intermediate state depend on the oxidation state^34^ – the pump acts as an energy ratchet. On the other hand, theoretical analysis of a model in which the same process is driven by a catalysed chemical reaction shows that the energy of the intermediate state is irrelevant and the sole determinant of the efficacy of pumping is chemical gating^34^ – the motor functions as an information ratchet.

The concept of a power stroke – a viscoelastic mechanical relaxation from a high energy state to a low energy state – is given in almost all textbooks of biochemistry and cell biology to explain how molecular machines such as myosin, kinesin, and the flagellar motor convert chemical energy (*e.g.* ATP hydrolysis) or osmotic energy (*e.g.*, a proton gradient) into mechanical motion and the performance of work. The recently synthesized molecular motor driven by chemical catalysis,^8^ and the concomitant theoretical analysis, shows unequivocally that this mechanism is incorrect. Strain (or other energetic interactions between components) stored in a molecular motor, while of the essence for light driven motors and for motors driven by external modulation of the chemical environment, is irrelevant for molecular motors driven by chemical catalysis, including ion transport.

In the molecular world, equilibrium is a dynamic state in which all possible motions are explored with equal probability in the forward and reverse directions. The key principle for designing molecular motors that are driven by chemical catalysis is to use the chemical energy to selectively prevent unwanted motion, rather than to cause the desired motion. Any description of the effect of the catalysed reaction in terms of “deposition of chemical energy” or as causing violent kicks or judo throws is wrong and highly misleading.

**Fig. 4:**
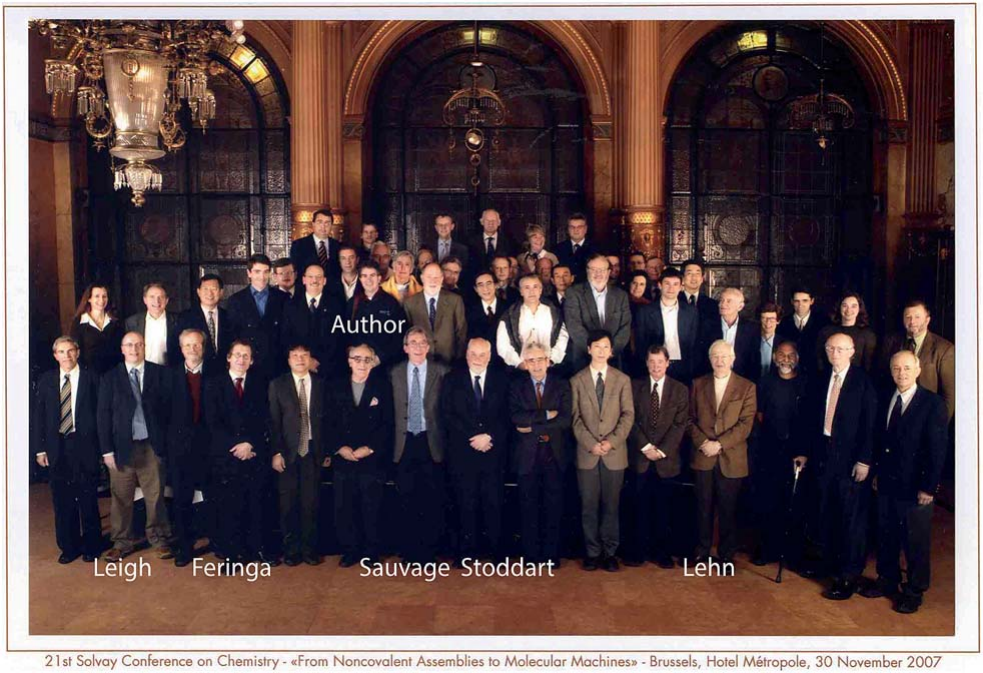
Participants of the 21^st^ Solvay Conference on Chemistry “From Noncovalent Assemblies to Molecular Machines”, Brussels, Hotel Metropole, 30 November 2007.

The talk given by Richard Feynman in 1959, “Plenty of room at the bottom”, represents a defining moment in the quest for making molecular motors, as does the 21^st^ Solvay Conference on Chemistry held in Brussels in 2007 (see Fig. 4). At this meeting there was much emphasis on how chemists are inspired by the fabulous machinery of biology, and how, in turn, the synthetic molecular analogues are teaching much about the fundamental principles by which bio-molecular machines function. A very important philosophy, beautifully reflected in the work on molecular motors, is contained in a phrase found on Feynman's blackboard at the time of his death – “What I cannot create I do not understand”. The Nobel laureates in Chemistry for 2016, and others, have provided techniques by which to create and tinker with molecular motors. This breakthrough is not only the precursor to what promises to be an amazing technology, the applications of which have yet to be developed, but also the means by which to come to penetrating and detailed understanding of the fundamental principles by which the molecular motors of life carry out their essential functions.
